# *SOX11* expression as a MRD molecular marker for MCL in comparison with t(11;14) and IGH rearrangement

**DOI:** 10.1007/s12032-018-1111-x

**Published:** 2018-03-08

**Authors:** Małgorzata Szostakowska, Michał Szymczyk, Kalina Badowska, Barbara Tudek, Anna Fabisiewicz

**Affiliations:** 1 Faculty of Biology, University of Warsaw, Miecznikowa 1, 02-096 Warsaw, Poland; 2 Department of Molecular and Translational Oncology, Maria Skłodowska-Curie Institute - Oncology Center, Roentgena 5, 02-781 Warsaw, Poland; 3 Department of Lymphoproliferative Diseases, Maria Skłodowska-Curie Institute - Oncology Center, Roentgena 5, 02-781 Warsaw, Poland; 4 Institute of Biochemistry and Biophysics, Polish Academy of Sciences, Pawińskiego 5A, 02-106 Warsaw, Poland

**Keywords:** Minimal residual disease, Mantle cell lymphoma, SOX11, Molecular marker

## Abstract

The main cause of death in mantle cell lymphoma (MCL) patients is relapse due to undetermined minimal residual disease (MRD) and therefore monitoring MRD is crucial for making the best treatment decisions. The gold standard method for MRD analysis is the quantitative polymerase chain reaction. The most commonly used molecular markers for measuring MRD in MCL are: t(11;14)(q13;p32) translocation or *CCND1* expression and IGH rearrangement. Such markers can, however, be found in other B cell non-Hodgkin lymphomas. Recent studies demonstrate that *SOX11* expression is highly specific for MCL and could be used as a marker for measuring MRD. Moreover, evidence shows that *SOX11* level could be predictive for overall survival (OS) and progression-free survival (PFS). We have measured MRD level in follow-up samples from 27 patients diagnosed with MCL using the molecular markers: t(11;14), IGH rearrangement and *SOX11* expression. We compared all markers by their sensitivity, utility and quantitative range. We also examined the predictive value of *SOX11* expression for OS and PFS. *SOX11* expression was found to have better specificity, quantitative range and utility than the t(11;14). The predictive value of *SOX11* expression was confirmed. At diagnosis, patients with high *SOX11* expression had shorter PFS than patients with low *SOX11* expression (*p* = 0.04*); differences between OS being statistically insignificant. To our best knowledge this is a first study comparing *SOX11* with t(11;14) and IGH rearrangement as markers of MRD level. Moreover, in this study we confirmed that *SOX11* is useful in cases when other molecular markers cannot be used.

## Introduction

Mantle cell lymphoma (MCL) is a rare and incurable disease (5–8% of all NHL), mostly appearing in males (3:1 males: females) at a median age of 68 years [1]. MCL is characterized by an aggressive course and multiple relapsing. According to the new World Health Organization (WHO) classification, there are two separate types of MCL:

- *Classic MCL* with IGH-unmutated B cells and *SOX11* expression, which usually follows an aggressive course and can transform to the very aggressive blastoid MCL with activation of certain molecular factors [2].
- *Leukemic non-nodal MCL* with IGH-mutated B cells, without *SOX11* expression, usually having an indolent outcome, but can transform to nodal aggressive MCL with activation of certain molecular factors (e.g., TP53) [2].

Relapse due to undetermined minimal residual disease (MRD) is the main cause of death in MCL patients, thereby making monitoring of MRD crucial. Recent studies have shown that consistent monitoring of MRD can improve treatment decisions [3–7]. MRD level can be also predictive for progression-free survival (PFS) [4–6]. In the MCL Younger trial, MRD positivity before autologous stem cell transplantation (ASCT) was highly correlated with shorter PFS [3]. Moreover, in patients being in clinical remission, MRD status estimated in peripheral blood (PB) was prognostic for overall survival (OS) [7]. Therefore, consistent monitoring of MRD in MCL could allow improvements to current treatments and predict clinical relapse, PFS and OS.

The gold standard method for measuring minimal residual disease is the quantitative polymerase chain reaction (qPCR). The most widely used molecular markers for measuring MRD in MCL are: t(11;14)(q13;p32) translocation or *CCND1* expression [8] and IGH rearrangement [9].

The t(11;14)(q13;p32) translocation which causes cyclin D1 (*CCND1*) overexpression can be detected in 90% MCL cases. CCND1 is one of the proteins that regulates cell cycle thus its upregulation drives B cells to proliferation [10]. It is applied either to diagnostics or to MRD monitoring for MCL [8]. A lack of this overexpression is characterized as a *CCND1* negative subtype of MCL [11] and appears in 10% of MCL cases. Moreover, cyclin D1 overexpression is also found in CLL [12], which has a similar clinical image to leukemic non-nodal MCL. The t(11;14) translocation is confirmed by fluorescence in situ hybridization (FISH) in 90% patients, but it is detectable by PCR methods in only 25–40% of cases [13].

IGH rearrangement is highly specific to each patient. It is used as molecular marker in many B cell neoplasms and allows detection of tumor cells in 80–90% of patients [14]. However, measuring MRD by IGH rearrangement is labor-intensive. It is based on the qPCR assay which demands designing allele-specific oligonucleotide (ASO) PCR primers. Furthermore, it is impossible to distinguish two subtypes of B cell neoplasms by this method in rare cases of composite lymphoma (CL).

Considering these aforementioned limitations, there is a need to find a specific and sensitive molecular marker for monitoring minimal residual disease in MCL. Recent studies have shown that *SOX11* expression is highly specific for MCL [15] and independent of the presence of t(11;14) [16] and thus it can be used as a molecular marker even for the *CCND1(*-*)* negative MCL subtype [11]. *SOX11* expression is also more specific for MCL than *CCND1* overexpression; being, respectively, found in 90–95% and 90% of MCL cases [17]. *SOX11* expression also distinguishes two types of MCL: classic from leukemic non-nodal [2] and can be predictive for OS [18, 19]. Evidence has shown that *SOX11* expression can be used as a measurement of MRD in MCL [20] with at least the same sensitivity but better specificity than t(11;14) [21]. Therefore, *SOX11* could potentially appear as a better molecular marker than t(11;14). In this study, we compared *SOX11* expression, t(11;14) and IGH rearrangement as MRD molecular markers to determine if *SOX11* can be used with at least the same sensitivity and specificity as other molecular markers.

## Materials and methods

PB and BM from 34 patients treated in the Department of Lymphoproliferative Diseases, Maria Skłodowska-Curie Institute—Oncology Center were collected: 71 samples at diagnosis and 254 samples during treatment. There were at least two samples from every patient at diagnosis and 3–15 samples were collected from 27 patients during treatment. PB samples from healthy donors served as negative controls. White blood cells (WBC) were isolated by density gradient centrifugation. Samples containing 5 × 10^6^ cells were frozen and stored at − 80 °C. The research protocol was reviewed and approved by the Ethics Committee of Maria Skłodowska-Curie Institute—Oncology Center. Informed consent was obtained from all individual participants included in the study before the collection of biological material.

RNA isolation from cell precipitate was performed with the GeneMATRIX Universal Purification Kit (EURx, Poland) according to the manufacturer’s protocol. RNA concentration and purity were measured using the NanoDrop spectrophotometer (ThermoFisher) and FlashGel system (Lonza). A 500 ng of RNA was reverse transcribed (RT) using the NG dART Reverse Transcription Kit (EURx) according to the manufacturer’s protocol. cDNA quality was estimated by PCR with GAPDH primers: F: 5′-GAAGGTGAAGGTCGGAGTC-3′; R: 5′-GAAGATGGTGATGGGATTTC-3′, using the NG dART RT-PCR kit (EURx) according to the manufacturer’s protocol. cDNA was diluted to a final volume of 35 µl when RT efficiency was low, and to a final 50 µl volume when RT efficiency was high. Diluted cDNA was stored at − 20 °C or +4 °C if used within 24 h. qPCR was performed according to a highly sensitive and specific qPCR assay for *SOX11* measurement previously described by Hamborg et al. (2012) [20], using the SensilFAST Probe Lo-ROX Mix (BioLine) without the uracil-DNA glycosylase (UNG) enzyme.

DNA isolation was performed using the Sherlock AX isolation kit (A&A Biotechnology) according to the manufacturer’s protocol. For IGH rearrangement, PCR and qPCR reactions were performed according to the BIOMED-2 Concerted Action BMH4-CT98-3936 protocol [9] using 7500 Fast Real Time PCR System (Applied Biosystems, Foster City, CA).

Standard curves and follow-up analysis were done according to van der Velden et al. (2007) [22]. PCR and qPCR reactions for t(11;14) were performed according to Pott et al. (2013) [23] For four patients, there was a need to design ASO PCR primers.

Sensitivity of molecular markers was defined by the lowest possible dilution detected by qPCR. The quantitative measurement range was set up as the lowest detected dilution which had a Ct lower than that of the negative controls background by three cycles.

MRD level was calculated to allow comparisons between molecular markers to be made. Results from diagnostic samples were set to a 1 (100%) MRD level while MRD level in follow-up samples was calculated as derived proportions.

Statistical analysis was performed on Microsoft-Excel using correlation, linear regression and log-rank testing. Graphs were constructed via GraphPad Prism software (GraphPad Prism Inc.).

## Results

*SOX11* expression was measured as a proportion of SOX11 copies/1000 reference genes (RG) copies. Results obtained for patients were very highly variable; from 2 × 10^−2^ *SOX11*/1000 RG to 353 *SOX11*/1000 RG. Four patients were diagnosed with blastoid MCL and all four had *SOX11* expression higher than 100 *SOX11*/1000 RG. Eighteen patients with low *SOX11* expression in the diagnostic sample were usually diagnosed with less aggressive disease. Three patients with low *SOX11* expression in the diagnostic sample diagnosed with aggressive advanced nodal MCL usually had multiple relapses during treatment. Sixteen patients with less than 1 *SOX11*/1000 RG were excluded from further analysis of MRD.

MRD analysis was performed for: 18 patients (53%) by *SOX11* expression, 9 patients (26%) by t(11;14) and 21 patients (61%) using IGH rearrangement. Due to low *SOX11* expression and poor sensitivity of the t(11;14) translocation, in 5 patients (15%) the only molecular marker that could be used was IGH rearrangement. For two patients with a high polyclonal background and low *SOX11* expression, the only molecular marker applicable was t(11;14). Because of the polyclonal background and poor sensitivity of t(11;14) in one patient (2%), the only molecular marker that could be used was *SOX11* expression.

**Fig. 1:**
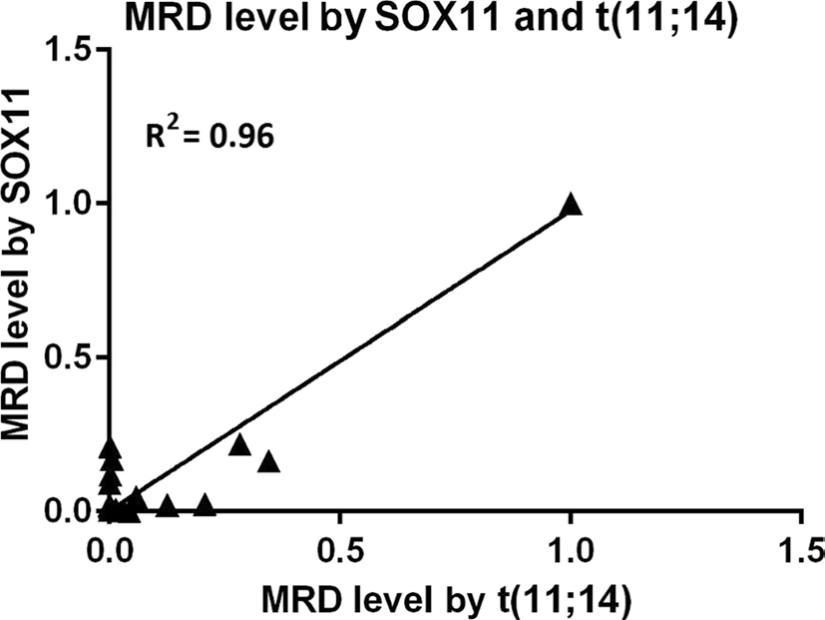
Linear regression of MRD levels measured by molecular markers of *SOX11* expression and t(11;14) translocation

Correlation between the MRD level measured by t(11;14) and *SOX11* expression was very high, at 0.98. Sensitivity for both methods was near 1 × 10^−5^ but for t(11;14) the mean quantitative measurement range was 1.2 × 10^−4^. The difference between MRD level estimated by both markers was not statistically significant, *p* > 0.98. For further analysis, we estimated a linear regression model using MRD level measured by *SOX11* expression and t(11;14) (Fig. 1).

**Fig. 2:**
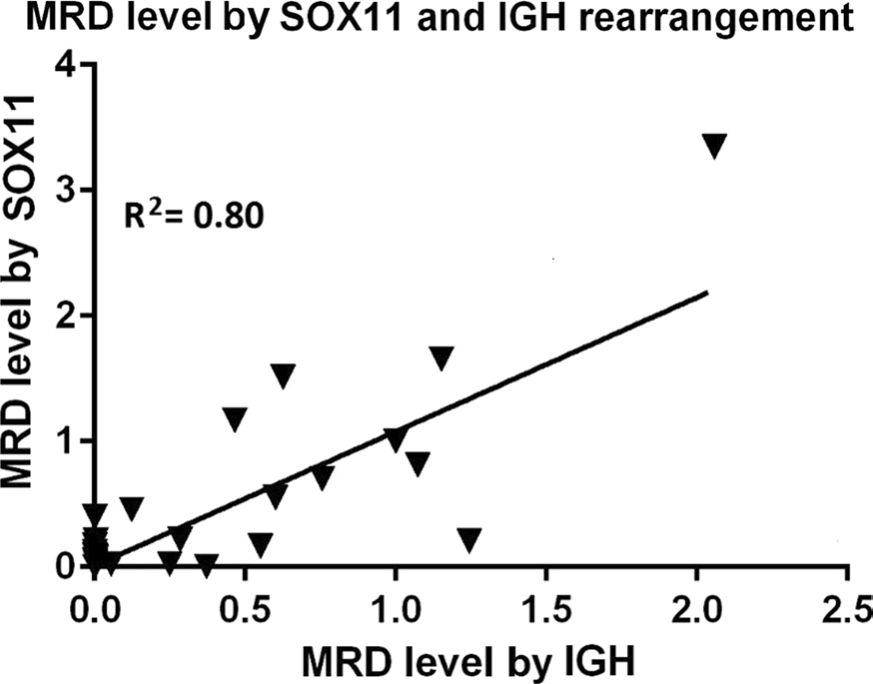
Linear regression of MRD levels measured by molecular markers of *SOX11* expression and IGH rearrangement

When MRD levels were measured in parallel by *SOX11* expression and the IGH rearrangement the correlation was also high at 0.89. The sensitivity of both molecular markers was near 1 × 10^−5^ and for IGH rearrangement the mean quantitative range was 5 × 10^−5^. As in the previous comparison, the difference between MRD level measured by both markers was not statistically significant, *p* > 0.79. Likewise, as before a linear regression model was estimated using MRD level calculated by *SOX11* expression and IGH rearrangement (Fig. 2).

**Fig. 3:**
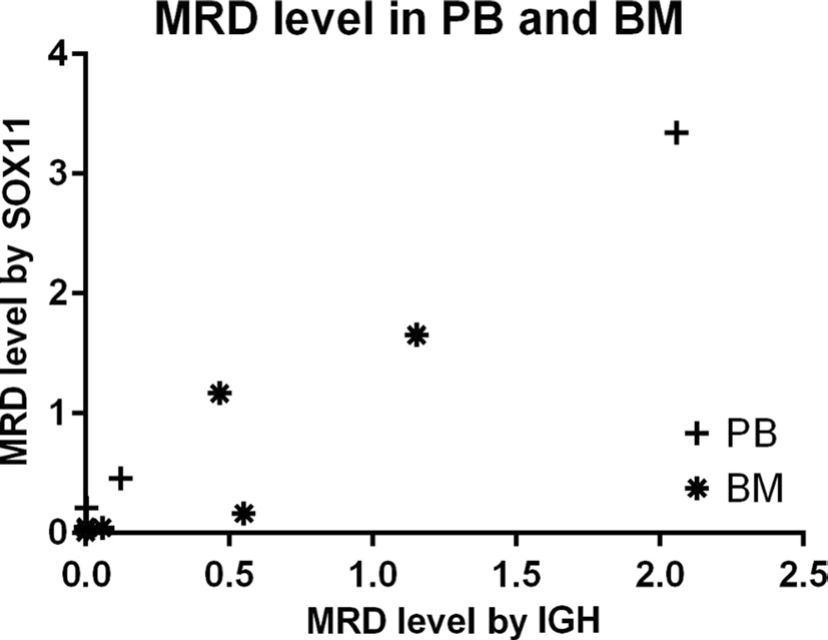
Comparison of MRD levels for PB and BM samples measured by the following molecular markers: *SOX11* expression, IGH rearrangement. BM results are different for both markers with an 87% similarity in trend

We also compared MRD level obtained from different biological material: PB and BM using the following molecular markers: *SOX11* expression and IGH rearrangement. Correlation between PB and BM samples was close at 0.85 for both molecular markers. (Fig. 3).

**Fig. 4:**
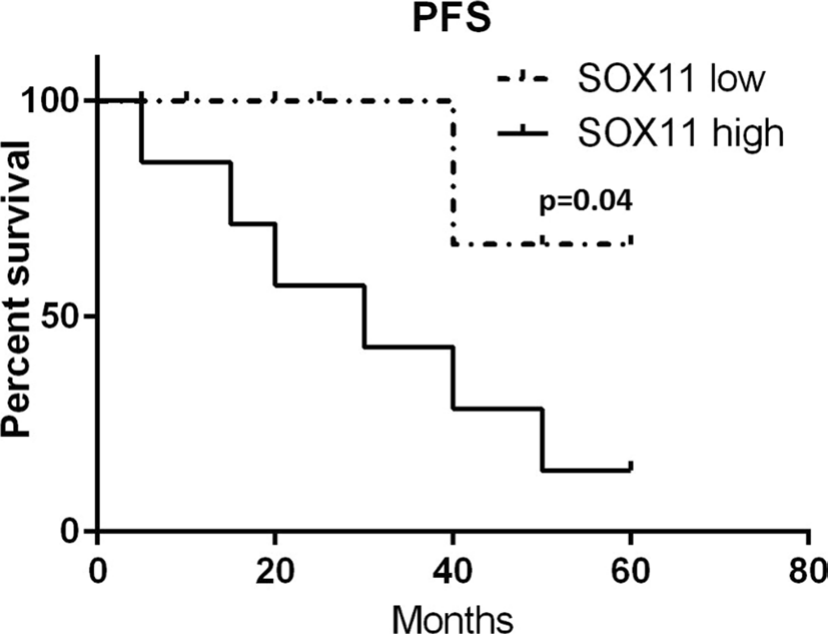
Progression-free survival in patients from two groups of *SOX11* expression: *SOX11*^negative/low^ − *SOX11* low and *SOX11*^intermediate/high^ − *SOX11* high. The difference between mean PFS in groups is statistically significant

**Fig. 5:**
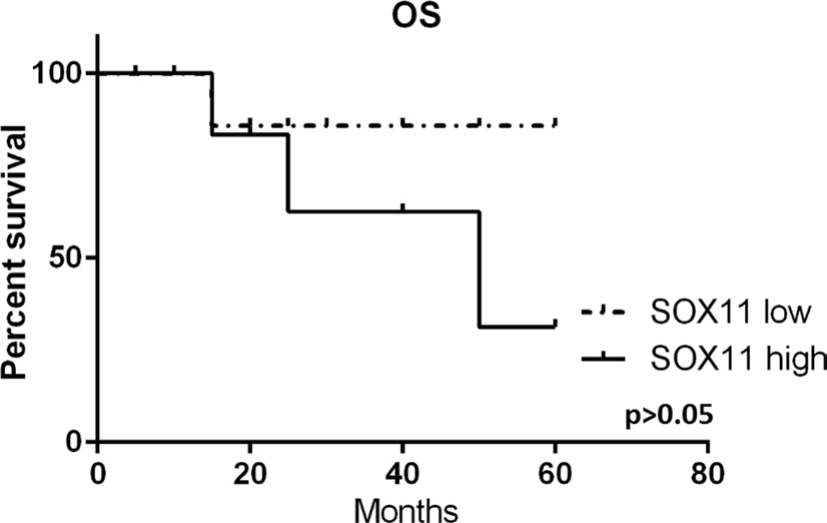
Overall survival in patients from two groups of *SOX11* expression: low and high expression of *SOX11*. The difference between OS is not statistically significant

In samples taken at diagnosis, *SOX11* expression was measured in 34 patients. Patients were divided into two groups: *SOX11*^negative/low^ with expression ≤ 10 *SOX11* copies/1000 RG copies and *SOX11*^intermediate/high^ with expression > 10 *SOX11* copies/1000 RG copies. Mean PFS in the *SOX11*^negative/low^ group of patients was ~ 36 months but for *SOX11*^intermediate/high^ it was only ~ 16 months (Fig. 4). The difference between mean PFS in the groups is statistically significant (*p* = 0.04). Mean OS in the *SOX11*^negative/low^ group of patients was ~ 41 months, and for the *SOX11*^intermediate/high^ group it was ~ 30 months; however, this difference between groups was statistically insignificant (*p* = 0.09) (Fig. 5).

## Discussion

Our study aimed to compare *SOX11* expression, t(11;14) translocation and IGH rearrangement as the qPCR molecular markers for measuring MRD level in MCL. We have demonstrated that all markers have similar sensitivity but the mean quantitative range for t(11;14) and for IGH rearrangement was lower than that for *SOX11* (1.2 × 10^−4^, 5 × 10^−5^, 1 × 10^−5^, respectively). For *SOX11* expression, the quantitative range was the same as the sensitivity, because there was no background from the negative control in any of the reactions. We have therefore concluded that *SOX11* expression is a more specific molecular marker than t(11;14) and IGH rearrangement. Moreover, there were no statistically significant differences between MRD level obtained using molecular markers: t(11;14) and *SOX11* or IGH rearrangement and *SOX11.*

MRD level measured by *SOX11* expression and t(11;14) translocation were comparable, with correlation of 0.98. We thus assumed that these molecular markers could be used interchangeably or that even *SOX11* expression can be used as a molecular marker instead of t(11;14). In this study, we applied the commonly used PCR primers designed for the major translocation cluster (MTC) which covers about 40% of MCL translocations. Insufficient specificity of t(11;14) primers used in qPCR reactions limits the use of this marker for monitoring MRD to only 25–40% cases [13]. Our study showed that the t(11;14) translocation was specific to 26% patients, while the *SOX11* expression to 53% patients, including one patient with *CCND1(*-*)* MCL. Our work confirms previous results suggesting that in comparison with the t(11;14) translocation, *SOX11* expression is a more specific molecular marker with similar sensitivity [20, 21].

Our work showed that MRD level measured by IGH rearrangement and *SOX11* expression was comparable, with a correlation coefficient 0.89. The IGH rearrangement was used as a MRD molecular marker for most patients (61%) compared to the other molecular markers: t(11;14) translocation and *SOX11* expression (26 and 53%, respectively). These results confirm previous assumptions about the utility of the IGH rearrangement [14]. Former work on comparing *SOX11* and IGH rearrangement suggests that *SOX11* expression could be less sensitive than IGH rearrangement [17]. In our study, *SOX11* expression was detectable at very low MRD level in the follow-up samples, especially for patients who had high *SOX11* expression at diagnosis (> 10 *SOX11* copies/1000 RG copies). However, for a few patients *SOX11* expression was not detectable in the follow-up samples at very low MRD level while IGH rearrangement was. All such patients had low *SOX11* expression at diagnosis (< 10 *SOX11* copies/1000 RG copies). Our results confirm previous conclusions, that *SOX11* has a lower sensitivity than IGH rearrangement [17], but only for patients with low *SOX11* expression at diagnosis. This may be caused by the fact that during treatment, the expression of *SOX11* decreases significantly. In patients with low baseline expression of *SOX11*, the level of expression during treatment decreases to values undetectable by RT-qPCR. However, in patients in whom the initial expression of *SOX11* is high, the decrease in the level of *SOX11* expression during treatment decreases within the limits of detection. Moreover, the difference in the sensitivity of these two markers may be due to the specification of the genetic material. RNA, which is the material for estimation of *SOX11* expression, is less stable thus, it degrades faster than DNA. Therefore, when a small amount of material is analyzed, the detectability of DNA is better than RNA.

Our study also investigated the suggested prognostic value [18, 19] of *SOX11* expression. We compared PFS and OS in groups of patients with low and high *SOX11* expression at diagnosis. Patients with high *SOX11* expression (> 10 *SOX11* copies/1000 RG copies) had shorter PFS than patients with low *SOX11* expression (< 1 *SOX11/*1000 RG). The difference between mean PFS in both groups was ~ 20 months and was statistically significant (*p* = 0.04). There was no statistically significant difference between OS for patients with high and low *SOX11* expression. Our results confirm the prognostic value of *SOX11* expression at diagnosis but only for progression-free survival which contrasts to previous studies in which low *SOX11* expression in vitro and in vivo was associated with poor prognosis, shorter OS and PFS [24, 25]. High expression of *SOX11* in tumor cells could lead to a more aggressive disease course [18, 19] with increased proliferation and cell survival, by activating FAK and CXCR4 [26]. Therefore, early detection of increasing level of *SOX11* expression may be important for improving therapeutic decisions.

Our results confirm high specificity of *SOX11* expression as a molecular marker for MRD in MCL. Moreover, the quantitative range of MRD level measured by *SOX11* was higher than the quantitative range for that obtained with the t(11;14) translocation and IGH rearrangement. Furthermore, MRD level obtained with the use of *SOX11*, t(11;14) and IGH rearrangement was highly correlated. We therefore hope that *SOX11* expression will be used as a molecular marker for measuring MRD in MCL with the same frequency of t(11;14) or IGH rearrangement. However, considering limitations emerging from stability of RNA, for the most efficient estimation of MRD level in MCL patients, *SOX11* expression should be used parallelly with IGH rearrangement. Simultaneous usage of both markers allows to confirm results obtained with *SOX11* expression. Moreover, this could permit to estimate MRD level in different subtypes of myelomas in rare cases of composite lymphoma. We also confirmed the prognostic value of *SOX11* expression, when high *SOX11* expression is correlated with poor prognosis. Considering the discordant results on the prognostic value of SOX11 expression, we think that this subject requires further study, including studies on pathways regulated by SOX11, *SOX11* methylation profiles and potential isoforms of SOX11.
